# Supplementary material for: A structural dynamics model for how CPEB3 binding to SUMO2 can regulate translational control in dendritic spines
Source: PLoS Comput Biol. 2022 Nov 8;18(11):e1010657. doi: 10.1371/journal.pcbi.1010657 (PMC9674179; doi:10.1371/journal.pcbi.1010657)
Supplement: S1 Text — Binding energy for SIM/SUMO2 complex. Additional potentials. Simulation methods: Equilibrum simulation of free RRMs; Equilibrum simulation of full length RBD; Structural prediction to the SUMO2/RBD complex; Free energy profiles for RNA dissociation; Free energy profiles for the closure motion of RRMs. Efficiency of the shift of RNA-binding equilibration. (PDF) [file pcbi.1010657.s001.pdf]

Supporting Information for: A structural dynamics model for how CPEB3 binding to SUMO2 can regulate translational control in dendritic spines

Xinyu Gu,[†,‡] Nicholas P. Schafer,[†,‡] Carlos Bueno,[†] Wei Lu,[†] and Peter G. Wolynes[*,†,‡]

†*Center for Theoretical Biological Physics, Rice University, Houston, Texas, US*

‡*Department of Chemistry, Rice University, Houston, Texas, US*

E-mail: pwolynes@rice.edu

Introduction to the AWSEM-3SPN2 force field

In this study, all simulations were carried out using the AWSEM(-3SPN2) force field. The AWSEM force field is a predictive coarse-grained model for proteins, whose Hamiltonian V_{AWSEM} is shown below,

$$V_{AWSEM} = V_{backbone} + V_{contact} + V_{burial} + V_{DH} + V_{HB} + V_{FM},$$

where $V_{backbone}$ is a peptide-like backbone term; $V_{contact}$ is a tertiary residual interaction term; V_{burial} is a many body burial term; V_{DH} is the Debye Huckel electrostatic term; V_{HB} is a beta-sheet favored, hydrogen bond term; V_{FM} is the associative fragment memory term, a bioinformatical term guiding the local-in-sequence interactions. In all simulations, we used the PDB structure 2N1W as the only memory for SUMO2 protein. We used the PDB structure 2MKJ to model the structure of free state CPEB3-RRMs and used the PDB structure 2M13 to model the partial structure of CPEB3-ZnF via Modeller. These two structures are then used as the only memories for RRMs and partial ZnF. The fragment memories for the rest of the ZnF were obtained from the known protein data bank using a sequence alignment algorithm.

For simulations containing the RNA fragment, we used the 3SPN2 force field which is a coarse grained model originally developed for simulating DNA, as the model for the RNA by regarding Uracil (U) as the base particle thymine (T) in 3SPN2:

$$V_{3SPN2} = V_{backbone} + V_{stacking} + V_{BP} + V_{CS} + V_{excl} + V_{DH},$$

where $V_{backbone}$ is a bonded term connecting 3-beads nucleotides backbone; $V_{stacking}$ is a stacking term between consecutive nucleotides; V_{BP} is a base pairing term between complementary nucleobases; V_{CS} is a cross stacking term between neighboring base pairs; V_{excl} is an exclusion term; V_{DH} is the Debye Huckel electrostatic term. The interaction between the protein and the nucleotides is modelled by an exclusion term and the Debye Huckel electrostatic term in AWSEM-3SPN2 force field. A more detailed introduction to the AWSEM-3SPN2 force field can be found in previous papers.[1–4] A debye length of 10 A is used for all Debye Huckel electrostatic terms. This value corresponds to a typical physiological solution condition when temperature T=300K and ionic strength equals 0.1 M, using a dielectric constant for water $\epsilon = 80$.[5] Original AWSEM-3SPN2 only assigns charges to the phosphate particle (-0.6) and four types of residues (Arg:+1, Lys:+1,

Asp:-1, and Glu:-1). Here additional charges were applied to the two zinc coordination sites include Zn1 (Cys654, Cys657, Cys681, Cys684) and Zn2 (Cys671, Cys676, His689, His697) to model the two zinc ions (+0.5 for each coordination residue) . The first nucleotide of the nucleic acid chains in 3SPN2 model lacks the phosphate particle, which is a proper approximation for very long nucleic acid chains. However, our simulations used a short RNA chain with a length of five nucleotides. Therefore, we assigned an additional charge of -0.6 to the sugar particle of the first nucleotide of our RNA chains. For the electrostatic interactions between protein and RNA, we modified the effective charge of each nucleotide into -1.0. Other parameters are set to the default values in AWSEM-3SPN2 (openmm version) and can be found at https://github.com/npschafer/openawsem and https://github.com/cabb99/open3spn2.

Binding energy for SIM/SUMO2 complex

The binding energy $E_{binding}$ for the SIM/SUMO2 complex is calculated using the equation:

$$E_{binding} = E_{SIM/SUMO2} - E_{SIM} - E_{SUMO2},$$

where $E_{SIM/SUMO2}$, E_{SIM}, E_{SUMO2} are the AWSEM energies for the SIM/SUMO complex, the SIM peptide and the SUMO2 protein separately. Here we used a complex structure (PDB ID: 6JXW) of a canonical SIM peptide (12 amino acids) and SUMO2 protein as the template and changed the sequence of the SIM peptide into the truncated CPEB3 sequence. The truncated CPEB3 sequence (12-amino acids) scanned over full length CPEB3 sequence with a 11-amino acids overlap between neighboring peptide sequences to calculate the binding energy of CPEB3 peptides with SUMO2 (S2 Fig).

Additional potentials

To model the two zinc ions coordinating with ZnF domain, we have assigned positive charges to coordination residues. However, this modification of charges can not take into account all the structural effects of coordinated zinc ions. To confine the structure of ZnF, we applied an AMHGo

potential (V_{AMHGo}) to all simulations containing the ZnF domain:

$$V_{AMHGo} = -k_{AMHGo} \sum_{ij} e^{-\frac{(r_{ij}^{A} - r_{ij}^{B})^2}{2\delta_{ij}^2}},$$

where $k_{AMHGo} = 0.3kcal/mol$ and other parameters are the same as corresponding parameters in Q value definition. Here structure A represents the ZnF structure during simulations and structure B is the Modeller structure of ZnF built from the PDB structure 2M13. We only considered the residue pairs with a distance closer than 8 A in the template structure.

To guide the formation of the inter-molecular beta sheet between RRM1-SIM and SUMO2-β2, we applied a spring potential (V_{rbias}) between these two groups of residues for a biased simulation:

$$V_{rbias} = \frac{1}{2} k_{rbias} (r - r_0)^2,$$

where $k_{rbias} = 0.1kcal/mol/A^2$, $r_0 = 15A$ and r is the distance between the mass centers of RRM1-SIM (P484-W489) and SUMO2-β2 (G27-R36).

To run the umbrella sampling simulations for studying the closure motion of RRMs, we used a biasing potential for the reaction coordinate Q_f:

$$V_{qbias} = \frac{1}{2} k_{qbias} (Q_f - Q_0)^2,$$

where $k_{qbias} = 10000kcal/mol$, Q_0 is the minimal for a simulation window and Q_f is the inter-domain Q value for current structure corresponding to the NMR structure of free RRMs.

To simulate the specific binding between CPEB3-RRMs and its target mRNA, we applied a sequence-specific contact term V_{ssc} between the protein and the RNA for all simulations containing RNA:

$$V_{ssc} = -k_{ssc} \sum_{ij} \gamma_{ij} e^{-\frac{(r_{ij} - r_{ij}^{B})^2}{2\delta^2}},$$

where $k_{ssc} = 0.8kcal/mol$, $\delta = 1.5A$, r_{ij}, r_{ij}^B are the distance between residues i and nucleotide j in current structure and the NMR structure of RNA-bound RRMs. γ_{ij} is the weight for each residue-nucleotide pair and equals $ln(C_{ij})$, where C_{ij} is the atomic contact number between residues i and nucleotide j in the NMR structure of RNA-bound RRMs. Here we used a distance threshold of 4.5 A to define atomic contacts and only considered residue-nucleotide pairs having no less than

14 atomic contacts in NMR structure ($C_{ij} \geq 14$). Given these parameter values, the RNA-binding affinity of RRMs calculated from our free energy profile ($\sim$ 8 kacl/mol) is comparable to the experimental value (6.4 $\sim$ 6.9 kcal/mol).

To calculate the free energy profiles of RNA dissociation for protein/RNA complex systems, we ran a two-dimentional umbrella sampling for each system. The major reaction coordinate is the value of V_{ssc}, corresponding to the biasing potential:

$$V_{sscbias} = \frac{1}{2} k_{sscbias} (V_{ssc} - V_0)^2,$$

where $k_{sscbias} = 1mol/kcal$ and V_0 is the minimal for a simulation window. For simulation windows with V_0 values close to 0, an additional biasing potential V_{Rbias} was applied to enhance sampling along the order parameter R, the distance between RNA and RNA-binding pocket in RRMs:

$$V_{Rbias} = \frac{1}{2} k_{Rbias} (R - R_0)^2,$$

where $k_{Rbias} = 0.1kcal/mol/A^2$, R_0 the minimal for a simulation window and R is the distance between the mass centers of RNA chain and RNA-binding pocket (F462, G465, P490, Y504, F506, K541, Q544, F570, K609, K645).

Simulation methods

All simulations were run in the openMM platform with the Langevin integrator at a constant temperature 300 K. We used 2 fs as the simulation time step and 1 ps as the damping time. For all umbrella sampling simulations, we output one sampling structure every 5000 steps.

Equilibrum simulation of free RRMs

We ran 20 independent equilibrium simulations of free RRMs, starting from the Modeller structure built from PDB structure 2MKJ. Each simulation is run for 6×10^6 steps and outputs one sampling structure every 10000 steps. All sampled structures in 20 trajectories were then used for the cartesian principal component analysis (PCA). We first superposed the beta sheet of RRM1 domain of each sampled structure with that of the initial structure, using several residues of the RRM1

beta sheet (V463-P468,E525,D526,Y530,Q544-P547) as targets. Then updated 3-dimentional coordinates of all the residues are recorded as PCA input data. The first principal component PC0 is then used as the order parameter to describe RRMs closure motion. The final frames of the 20 simulations are considered as predicted structures and were clustered into 3 subgroups (S3 Fig).

Equilibrum simulation of full length RBD

By fusing the ZnF domain to the C-terminal of the three representative RRMs structures shown in S3 Fig, we obtained three initial structures for full length RBD simulation. We ran 20 independent equilibrium simulations for each initial structure (60 simulations overall). Each simulation was run for 6×10^6 steps and the final frame is considered as the single predicted structure for full length RBD.

Structural prediction to the SUMO2/RBD complex

Using the NMR structure of SUMO1/CBP-ZnF complex (PDB ID: 2N1A) as a template, we can dock the SUMO2 protein to the ZnF of the 60 predicted structures of full length RBD and obtain 60 initial structures for the SUMO2/RBD complex structure prediction. For each initial structure, a biased AWSEM simulations with an additional potential V_{rbias} was run for 6×10^6 steps. After turning the V_{rbias} term off, the last frame of the biased simulation was relaxed for 2×10^7 steps. The final structures of the relaxed simulations were considered as candidates for further evaluation and selection.

Free energy profiles for RNA dissociation

For the RRMs/RNA system, we used the Modeller structure built from the NMR structure of RNA-bound RRMs (PDB ID: 2MKI) as the initial structure. The RNA sequence used in all simulations is also the same as the sequence in PDB structure 2MKI: 5'-CUUUA-3'. For the RBD/RNA system, we attached the ZnF domain to the C-terminal of RRMs in the RRMs/RNA Modeller structure to obtain the initial structure. For the SUMO2/RBD/RNA system, we first docked the RNA chain to the RNA-binding pocket in the predicted SUMO2/RBD complex. Then we ran a biased equilibrium simulation for 8×10^6 steps with the $V_{sccbias}$ term on. In this simulation, we set $k_{sscbias} = 5 mol/kcal$ and $V_0 = -35 kcal/mol$ so that the sequence specific protein/RNA contacts

were correctly formed in the final structure, which was then used as the initial structure for later umbrella sampling simulations.

For all these RNA-bound systems, we applied a 2D umbrella sampling method to archieve sufficient sampling using both V_{ssc} and R as two biasing reaction coordinates. The biasing potentials $V_{sscbias}$ and V_{Rbias} are mentioned above. We first turned off the V_{Rbias} term and set 33 simulation windows with V_0 evenly distributing from -35 kcal/mol to -3 kcal/mol. We also turned on the V_{Rbias} term and set 9 simulation windows with R_0 evenly distributing from 1 nm to 5 nm when V_0 equals -2, -1 or 0 kcal/mol. (So there are 60 simulation windows overall.) Each simulation lasts 8×10^6 steps. The first 1×10^6 steps are treated as equilibration and are then discarded in later analysis. The free energy profiles are calculated by 2D-WHAM analysis [6] and projected into another order parameter, PC0.

Free energy profiles for the closure motion of RRMs

For all the systems containing the RBD (including RBD, SUMO2/RBD shown in Fig 4 and the RBD/RNA, SUMO2/RBD/RNA shown in S5 Fig), we employed the umbrella sampling method to calculate free energy profiles for the closure motion of the two RRMs. For the SUMO2/RBD/RNA triple complex, we used the same initial structure as for the previous RNA dissociation simulations. For the other combinations, we simply removed unneeded components from that structure to build initial structures. The structural similarity to the NMR structure of free RRMs, Q_f was used as a biasing reaction coordinate. The biasing potential V_{qbias} is mentioned above. For each system, we set 71 simulation windows with Q_0 evenly distributing from 0.1 to 0.8. Each simulation lasts 8×10^6 steps. The first 1×10^6 steps are treated as equilibration and are then discarded in later analysis. Another order parameter, PC0 is used for 2D-WHAM analysis. [6] 1D free energy profiles along PC0 axis are shown in S6 Fig.

Efficiency of the shift of RNA-binding equilibration

1D Free energy profiles for RNA dissociation show that the difference of RNA-binding affinity of the SUMO2/RBD complex and that of the RBD by itself is around 2 kcal/mol. Therefore, an

exchange equation for RNA-binding can be written down:

$$m \cdot CPEB + CPEB_S \rightarrow m \cdot CPEB_S + CPEB, \qquad \Delta G = -2kcal/mol$$

where $CPEB$ and $CPEB_S$ represent CPEB3 and SUMOylated CPEB3, and the prefix "$m\cdot$" represents RNA-bound complex. In equilibrium, we have:

$$\frac{[m \cdot CPEB_S]}{[m \cdot CPEB]} = e^{-\Delta G/RT} \frac{[CPEB_S]}{[CPEB]}$$

Based on Western blot experiments on CPEB3[7] and Orb2[8] extracted from the brain, the fraction of SUMOylated CPEB3, $[CPEB_S]_W$, is comparable to that of deSUMOylated CPEB3, $[CPEB]_W$, in the basal state. However, the Western blot result shows only the sum of $[m\cdot CPEB_S]$ and $[CPEB_S]$ or the sum of $[m \cdot CPEB]$ and $[CPEB]$, since the extraction and purification process would wash out most associated RNAs from RNA binding proteins.[9] Here we assume that CPEB3, as an efficient translational regulator, has much higher concentration than its target mRNAs. Given this assumption, $[m \cdot CPEB_S]$ or $[m \cdot CPEB]$ is negligible in comparison with $[CPEB_S]$ or $[CPEB]$, and thereby $[CPEB_S] \simeq [CPEB_S]_W$, $[CPEB] \simeq [CPEB]_W$. Finally, we would find that the ratio of repressed target mRNA to active target mRNA, $\frac{[m\cdot CPEB_S]}{[m\cdot CPEB]}$, is approximately 30 in basal synapses.

References

1. Davtyan, A., Schafer, N. P., Zheng, W., Clementi, C., Wolynes, P. G., and Papoian, G. A. (2012) AWSEM-MD: protein structure prediction using coarse-grained physical potentials and bioinformatically based local structure biasing. *The Journal of Physical Chemistry B 116*, 8494–8503.

2. Hinckley, D. M., Freeman, G. S., Whitmer, J. K., and De Pablo, J. J. (2013) An experimentally-informed coarse-grained 3-site-per-nucleotide model of DNA: Structure, thermodynamics, and dynamics of hybridization. *The Journal of chemical physics 139*, 10B604_1.

3. Freeman, G. S., Hinckley, D. M., Lequieu, J. P., Whitmer, J. K., and De Pablo, J. J. (2014) Coarse-grained modeling of DNA curvature. *The Journal of chemical physics 141*, 10B615_1.

4. Lu, W., Bueno, C., Schafer, N. P., Moller, J., Jin, S., Chen, X., Chen, M., Gu, X., Davtyan, A., de Pablo, J. J., et al. (2021) OpenAWSEM with Open3SPN2: A fast, flexible, and accessible framework for large-scale coarse-grained biomolecular simulations. *PLoS computational biology 17*, e1008308.

5. Tsai, M.-Y., Zheng, W., Balamurugan, D., Schafer, N. P., Kim, B. L., Cheung, M. S., and Wolynes, P. G. (2016) Electrostatics, structure prediction, and the energy landscapes for protein folding and binding. *Protein Science 25*, 255–269.

6. Grossfield, A. WHAM: the weighted histogram analysis method. version 2.0.9.1; `http://membrane.urmc.rochester.edu/wordpress/?page_id=126`.

7. Drisaldi, B., Colnaghi, L., Fioriti, L., Rao, N., Myers, C., Snyder, A. M., Metzger, D. J., Tarasoff, J., Konstantinov, E., Fraser, P. E., et al. (2015) SUMOylation is an inhibitory constraint that regulates the prion-like aggregation and activity of CPEB3. *Cell reports 11*, 1694–1702.

8. Hervas, R., Rau, M. J., Park, Y., Zhang, W., Murzin, A. G., Fitzpatrick, J. A., Scheres, S. H., and Si, K. (2020) Cryo-EM structure of a neuronal functional amyloid implicated in memory persistence in Drosophila. *Science 367*, 1230–1234.

9. Baltz, A. G., Munschauer, M., Schwanhäusser, B., Vasile, A., Murakawa, Y., Schueler, M., Youngs, N., Penfold-Brown, D., Drew, K., Milek, M., et al. (2012) The mRNA-bound proteome and its global occupancy profile on protein-coding transcripts. *Molecular cell 46*, 674–690.
