# Supplementary material for: A structural dynamics model for how CPEB3 binding to SUMO2 can regulate translational control in dendritic spines
Source: PLoS Comput Biol. 2022 Nov 8;18(11):e1010657. doi: 10.1371/journal.pcbi.1010657 (PMC9674179; doi:10.1371/journal.pcbi.1010657)
Supplement: Attachment — Submitted filename: Response Letter.pdf [file pcbi.1010657.s013.pdf]

Response Letter

Dear Editor,

We sincerely thank the reviewers for their comments. We have made some revisions described below to address these comments. (All changes to the manuscript text are reproduced here and are annotated by highlighting). We are therefore now pleased to submit a revised manuscript titled "*A structural dynamics model for how CPEB3 binding to SUMO2 can regulate translational control in dendritic spines*", by Xinyu Gu, Nicholas P. Schafer, Carlos Bueno, Wei Lu and Peter G. Wolynes.

Reviewer 1

Gu and others built the models of RNA binding to CPEB3-RBD with and without the protein SUMO2. The results of coarse-grained AWSEM molecular dynamic simulations suggest the influence of SUMO2 on the RNA affinity as well as the dissociation pathway. Both the system and the designation of the simulations are interesting. However, I have a few comments about this manuscript.

Comments:

The authors state that they have calculated the free energy profile for RNA dissociation from the SUMO2/CPEB3-RBD complex. These calculations show that the RNA-binding free energy for the SUMO2/CPEB3-RBD complex is 2 kcal/mol larger than that for isolated RBD in deSUMOylated CPEB3 (Ref 22). Could the authors describe the difference between the systems in the two papers? It seems that these systems are similar.

Author reply:

We thank Reviewer 1 for this question. Ref. 23 (which we presume is the one Reviewer 1 intended to refer to) studies the isolated CPEB3-RBD interacting with RNA. We studied this exact construct also in this paper (please see the left panels of Fig. 6A and Fig. 6B). The sequences of the CPEB3-RBD domain are the same in Ref. 23 as our paper. Nevertheless, to understand the effect of SUMOylation, we needed to study the full SUMO2/CPEB3-RBD complex. This was not studied experimentally in Ref. 23 and to our knowledge no laboratory data exist for it. Thus we employ our predicted affinity for this complex in our discussions.

In this manuscript, the RBD structure is constructed via Modeller. In addition, the SUMO2/RBD complex is obtained by docking SUMO2 to the ZnF domain in the 60 structures of full-length RBD. In my opinion, the authors should provide the reliability of the MD model. Maybe there are no experimental affinity data of RNA to SUMO2/RBD. But I think some key interactions, binding poses, or some structural characteristics can be used to prove the MD simulation results.

Author reply:

We thank Reviewer 1 for these comments. It is true that the initial structure for the full-length RBD is constructed via Modeller and the initial structures of SUMO/CPEB3-RBD complex are obtained by docking SUMO2 to the ZnF domain in the 60 structures of full-length RBD. However, for every initial structure mentioned above, we have run a fairly long MD simulation to relax the system to the equilibrium state of our force field (Please see the details in SI). For the structural predictions to the SUMO/CPEB3-RBD complex, we have carried out cluster analysis and frustration analysis to select the final representative structure (Please see the details in Fig. 4). We understand the models we have presented here are not completely calibrated since there are no experimental affinity measurements of the SUMO2 to RBD and also no experimental affinity data of RNA to SUMO2/RBD. Our experience in other systems is that AWSEM does a good job on affinities. Nevertheless, these computationally built models do suggest a

potential mechanism underlying the switchable translational control in dendritic spines. We believe it is useful to share our novel computational results and insights with the broad audience of PLOS Computational Biology even without direct laboratory calibration and we look forward to experimental studies testing the specific structures and dynamical mechanism we proposed in this manuscript.

In section 2.2, the authors claim that "SUMO2, even after it has become covalently attached to CPEB3, can also non-covalently interact with the SUMO-Interacting Motif (SIM) in CPEB3." Could the authors state that in general SUMO2 is covalently or non-covalently attached to CPEB3?

Author reply:

We thank Reviewer 1 for this question. In general, SUMO2 is covalently attached to CPEB3 (as known as the SUMOylation of CPEB3), which has been reported in experimental work from Kandel and his colleagues (Ref. 12). The covalent SUMOylation site has been predicted to be located at the N-terminal of CPEB3 (Ref. 16). Nevertheless SUMO2 is also available to form (non-covalent) protein-protein interaction with CPEB3, even after it is covalently attached to CPEB3. Furthermore, the bioinformatic searches suggest that the RBD domain at the C-terminal of CPEB3 contains classical SIM motifs which are known to non-covalently interact with SUMO2.

<u>Reviewer 2</u>

The manuscript presents a computational study on the assembly of the CPEB3 protein to sumo and RNA. This is a complex and rich system due to its large size and high flexibility. It is much more complex than typical bimolecular reactions of protein-protein or protein-DNA. Its biological relevance is high. The manuscript is nicely written and the results are clearly presented.

Comments:

1. The study involves MD simulations using a simple model on a very complex system. Some calibration of the model is performed. This is unique and appreciated. The discussion on the experimental interpretation is not clear enough and should be better communicated. It is hard to tell if the manuscript is addressed to a computational community of modeling or to the biological community who is interested in CPEB3. It will be better if it will be addressed to both communities. For example, the last section seems to be very different than the rest of the manuscript. The integration between the different parts may improve the clarity of the manuscript.

Author reply:

We thank Reviewer 2 for these comments. We understand that our introduction and discussion sections which address the system biology of CPEB3 are very different in structure from the results and methods sections which present primarily our findings based on MD simulations. We note, however, that the motivation of all our MD simulations has been to fill in the missing pieces in the system biology of CPEB3 -- how the translational activation from the "permanently" stable CPEB3 prion can be switched off to a new basal state when synapses go back to that basal state. In the discussion section, we have clearly laid out the way our MD simulation results on the RNA binding affinity explain that there is an RNA binding shift in basal synapses and how this conclusion allows us complete the system biology picture for CPEB3 (Please see Fig. 8B for details.) We believe our novel simulation results and the potential mechanisms implied by them will be of fundamental interest to both the neuroscience and the molecular biology communities and, in any event, provide insights for further experimental and computational studies that pursue the mechanism of the coupling of CPEB3 functions with the formation of long-term memory. We believe our work also provides a paradigm example whereby using computational methods can connect otherwise cryptic clues from experiments so as to address biological problems at the systems level.

2. For example, a schematic figure that supports the intro may assist in clarifying the details. Perhaps some parts of the last figure can be moved to an introductory figure.

Author reply:

We thank Reviewer 2 for this suggestion. To clarify the details of the background and our motivation, we have added Figure 1 and its caption (page 3) in our revised manuscript as below:

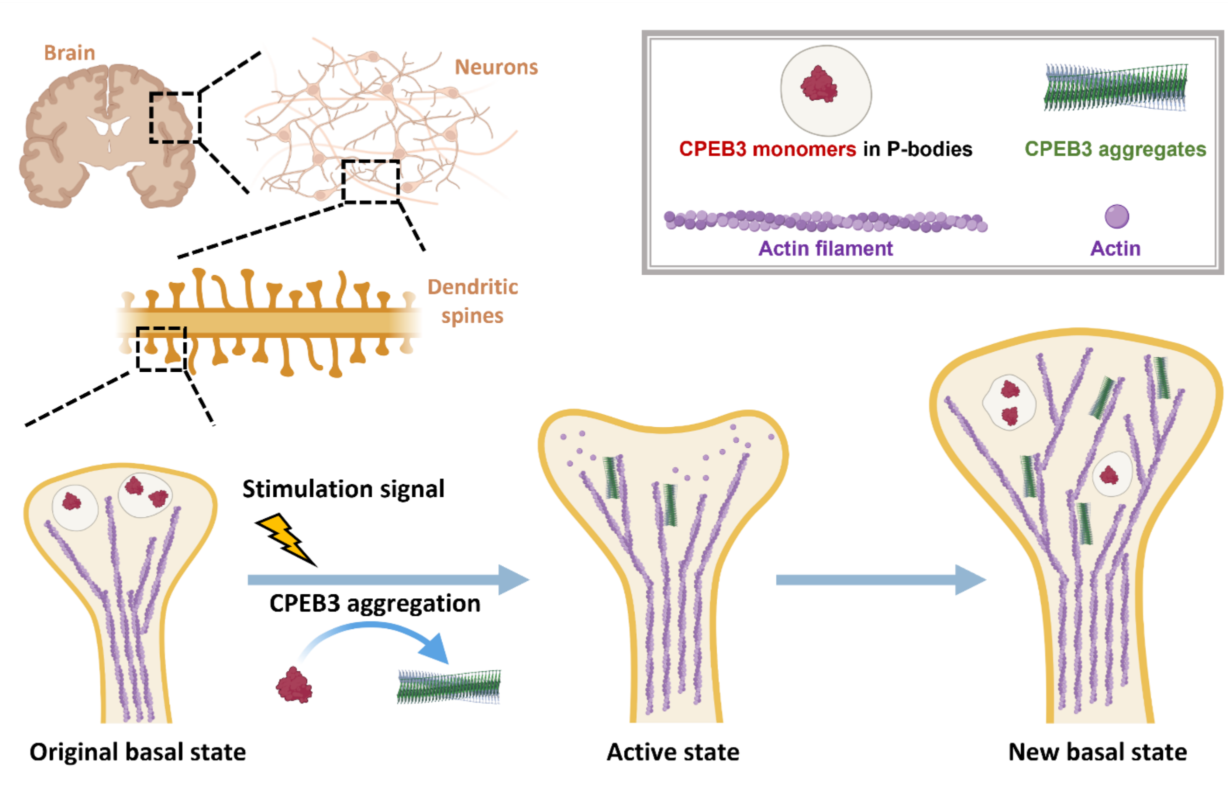

Figure 1. The conformational changes of CPEB3 provide a mechanism for the switchable translational control in dendritic spines. In a basal state, monomeric CPEB3 (shown in red) is colocalized with P-bodies and represses the translation of its target mRNAs. In response to a stimulation signal, CPEB3 monomers are released from P-bodies and then aggregate into CPEB3 fibers (shown in green). CPEB3 fibers (using the vectorial channeling mechanism) activate the local translation of synaptic proteins, including the actin proteins which are the molecular basis for the growth of the spines. The stability of the remaining prion-like CPEB3 aggregates would raise a problem of how the translational activation is turned down after a stimulation pulse. SUMO binding provides a route to return to the new basal state. A legend is shown at the upper-right corner.

3. A "conclusions" section is missing. Also the Discussion part includes only a single subsection? Was it supposed to include an additional part? Is the submitted manuscript complete?

Author reply:

We thank Reviewer 2 for the double-check. The manuscript is complete. Our "*Discussion*" section represents as "*Conclusion*" section in general. The main results are all included in the "*Results*" section and we do not repeat them.

4. How was the RNA modeled? Is there anything that differentiate the RNA modeling from DNA models?

Author reply:

We thank Reviewer 2 for this question. The RNA chains in our systems are very short, only containing 5 nucleotides. So in our case, the RNA chains do not form specific secondary structures and would perform similarly as single chain DNAs. Therefore, we adopted a modified coarse-grained DNA model, the 3SPN2 model, for the RNA chains in our systems. A Go potential is added to simulate the sequence-specific protein-RNA interactions and is calibrated based on the experimental RNA binding affinity. (As described are the details in the SI.)

5. Why calibrating the affinity was done to 8kcal/mol while the exp value is closer to 6 kcal/mol?

Author reply:

We thank Reviewer 2 for this question. The measured binding affinity value is 6.4~6.9 kcal/mol. We believe that our result is sufficiently close to provide an understanding of the qualitative differences in binding affinity. Calibrating the binding affinity more precisely to the experimental data is very unlikely to modify the story which is based on structural conflicts, primarily.

6. A sentence that explains what Sumo2 is relative to sumo, might be helpful.

Author reply:

We thank Reviewer 2 for this suggestion. SUMO2 is one specific homolog in the SUMO protein family. Ref. 12 has reported that CPEB3 can be SUMOylated by SUMO2 and SUMO2 mRNAs are one of CPEB3's targets. For clarity, we have rephrased the third paragraph (page 4) of the introduction section in the revised manuscript:

>>

One possibility that has been suggested involves the SUMOylation of CPEB3. There is evidence that SUMOylation, a reversible post-translational modification, can regulate CPEB3 function. SUMO proteins, small ubiquitin-like modifier proteins, can be covalently attached to lysine residues of target proteins. After such SUMOylation, the SUMO modification can subsequently be deconjugated (deSUMOylation). Monomeric CPEB3 is found to be SUMOylated by SUMO2, one homolog of the SUMO protein family, and in that form it is soluble in basal synapses. After stimulation, CPEB3 becomes deSUMOylated and aggregates.(12) SUMOylation of CPEB3 has been found to facilitate its colocalization to P bodies and is crucial for repressing translation.(13) DeSUMOylation of CPEB3 exposes the prion-like domain (PRD) and the actin-binding domain (ABD) of CPEB3 and triggers actin-facilitated CPEB3 aggregation as a result.(16) An interesting fact in thinking about the system biology of SUMO2/CPEB3 is that SUMO2 mRNA itself is one of CPEB3's targets. Therefore, there is a negative feedback loop between CPEB3 aggregation and SUMO2 synthesis: (12) The CPEB3 prion activates the translation of SUMO2 which can then be used for CPEB3 SUMOylation and lead to a return to translational repression. This negative feedback loop could thereby serve as a control on the balance between activation and repression of translation by CPEB3.

<<

Reviewer 3

This manuscript is part of an ongoing effort from Dr. Wolynes and colleagues to provide insights into an important problem via modelling: what are the consequences of conversion of CPEB proteins from monomer to aggregates in the synapse and implications in stabilization of memory. In this manuscript, through modelling, they attempted to address how translation activation by aggregated CPEB3 protein can be prevented from running away. This is

important since the aggregates are supposed to be self-sustaining. The model takes into consideration three factors: CPEB3 binds mRNA, SUMOylation prevents CPEB3 aggregation and promotes p-body formation, and aggregated CPEB3 translates SUMO2. The major conclusion from the modelling is that the binding of SUMOylated monomeric CPEB3 to mRNA is stronger than that of deSUMOylated aggregated CPEB3. The model suggests aggregated CPEB3, by translating SUMO2, can restrict its own activity. Controlling persistent translation activation by translating a modifier although not entirely novel, nonetheless an important insight. However, the authors should consider and discuss following issues:

Comments:

1) What is the relationship between stronger mRNA binding and P-body formation and/or translation inactivation? If SUMOylation is the major driver for p-body formation and P-body prevents translation, then what is the contribution of stronger mRNA binding. It is also unclear why a weaker mRNA binding would promote translation. In fact, stronger mRNA binding can promote translation.

Author reply:

We thank Reviewer 3 for these questions. It is a little bit confusing for us to argue that "stronger mRNA binding can promote translation", without discussing what mRNA is binding with. If mRNA is strongly binding with a translational repressor, its translation would obviously be repressed. The P-body, as one type of cytoplasmic membrane-less granule, is comprised primarily of mRNAs in complex with proteins associated with translation repression and 5′-to-3′ mRNA decay (Luo, et. al. *Biochemistry* **2018** 57 (17), 2424-2431). In our case, stronger mRNA binding with SUMOylated CPEB3 in P-bodies, would facilitate the recruitment of active mRNAs to the P-bodies and the subsequent silencing of those mRNAs. As for why CPEB3 aggregates promote translation, the introduction section has briefly mentioned a few of the mechanisms proposed up-to-date (Please see the details in Ref 13-15).

2) The model is based on published experimental observations. However, all published studies of CPEB3 utilizes in-cell translation to understand the relationship between CPEB3-SUMolytaion, aggregation and translation. Since several of these experiments were done in the background of endogenous unmodified CPEB3 in presence of other regulators of translation and aggregation, it remains unclear whether small fraction of SUMOlyated CPEB3 or bulk-SUMOlyation is responsible for the translation and aggregation differences. These two scenarios would invoke different models.

Author reply:

We thank Reviewer 3 for these comments. We agree with Reviewer 3 that many of published studies were done in the background of endogenous CPEB3 in cell. However, in-vitro translation experiments with different formats of CPEB (Fig. 3E in Ref. 14) and in-vitro detergent insolubility experiments on SUMOylated CPEB3 and deSUMOylated CPEB3 (Fig. 3 in Ref. 12) have also been conducted to consolidate those in-cell experimental observations. We do agree with Reviewer 3 that cleaner experiments would help further refine the models of CPEB3 function. However, we are a theoretical and computational research group. We do not have the appropriate experimental capabilities in our group. We hope our novel computational results and insights will intrigue the experimentalists in the CPEB community so that computation and experiment could support each other and achieve a better understanding of the role of CPEBs in synaptic plasticity.

3) Finally, the aggregation of CPEB3 in a specific synapse supposedly has two consequences: transition from repression to activation and maintaining an altered state of the synapse. The model shows aggregation provides a

transient burst of activation and SUMOylation returns the process to the initial state of the synapse. This is compatible with the transition from repression to activation, but it does not accommodate the second consequence. If it returns to the original basal state, then aggregation-mediated translation would not contribute the maintenance of the modified state of the synapse. Or am I missing something important here? Anyway, this needs to be clarified in the discussion.

Author reply:

We thank Reviewer 3 for these questions. In fact, we have now clarified that the synapses do not return to the original basal state, but rather return to a new basal state. As illustrated in Fig. 8C, the level of CPEB3 fibers increases after each stimulation pulse in order reach to a new basal state each time. To emphasize and clarify this point, we also added a new figure to the introduction section (Fig 1 in page 3).
